# Nonionic Surfactant Vesicles in Ocular Delivery: Innovative Approaches and Perspectives

**DOI:** 10.1155/2014/263604

**Published:** 2014-06-03

**Authors:** Ranjan Ku. Sahoo, Nikhil Biswas, Arijit Guha, Nityananda Sahoo, Ketousetuo Kuotsu

**Affiliations:** Department of Pharmaceutical Technology, Jadavpur University, Kolkata 700032, India

## Abstract

With the recent advancement in the field of ocular therapy, drug delivery approaches have been elevated to a new concept in terms of nonionic surfactant vesicles (NSVs), that is, the ability to deliver the therapeutic agent to a patient in a staggered profile. However the major drawbacks of the conventional drug delivery system like lacking of permeability through ocular barrier and poor bioavailability of water soluble drugs have been overcome by the emergence of NSVs. The drug loaded NSVs (DNSVs) can be fabricated by simple and cost-effective techniques with improved physical stability and enhance bioavailability without blurring the vision. The increasing research interest surrounding this delivery system has widened the areas of pharmaceutics in particular with many more subdisciplines expected to coexist in the near future. This review gives a comprehensive emphasis on NSVs considerations, formulation approaches, physicochemical properties, fabrication techniques, and therapeutic significances of NSVs in the field of ocular delivery and also addresses the future development of modified NSVs.

## 1. Introduction

**Figure 1:**
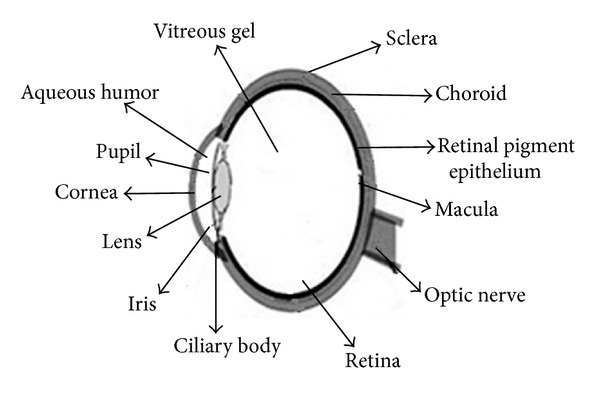
Structure of eye.

The body barriers like dynamic, tissue, and ocular blood barriers have presented major challenges to the formulation scientists and pharmacologists in the development of ocular drug delivery for decades. In terms of drug delivery, the eye can be considered to have four target sites: (i) the preocular structures of the front of the eye (e.g., conjunctiva and eyelids); (ii) the cornea; (iii) the anterior and posterior chamber and associated tissues; and (iv) posterior eye segment (e.g., retina and vitreous cavity) (Figure 1). Topical, systemic, periocular, and intravitreal are common routes of drug administration for the treatment of eye disorders and infections. Topical instillation is the most widely preferred noninvasive route of drug administration to treat diseases affecting the anterior segment. Conventional eye drops account for 90% of the marketed ophthalmic formulations. The reason may be attributed to ease of administration and patient compliance [1, 2]. However, in topical drop administration the ocular bioavailability is very low and extensive precorneal loss is caused by tear turnover, nasolacrimal drainage, reflex blinking, and ocular static and dynamic barriers [3, 4]. Hence, about 1-2% of the drug penetrates cornea and reaches the intraocular tissues after instillation of an eye drop while major portions are absorbed systemically [5, 6]. Due to the presence of blood-aqueous barrier and blood-retinal barrier, systemic administration leads to accumulation of high loading dose at target site which results in unavoidable systemic side effects like stomach upset and disturbed gastrointestinal motility.

In order to overcome the problems of conventional ocular therapy, such as short residence time, impermeability of corneal epithelium, and frequent instillation and ocular drug delivery barriers, numerous nanocarriers have been developed. Many of the ocular drug delivery systems (e.g., liposomes, micelles, solid lipid, and polymer-based nanoparticles) have reached the late stages of development, and some of them were approved but due to blurred vision or lack of patient compliance, they have not been universally accepted. As a result, drug delivery system has been enriched by the introduction of novel vesicles which improved both permeability and bioavailability of poorly water soluble drugs [7]. Inspired by the unique properties of NSVs, our review expands on the versatility and flexibility of NSVs and how such nanostructures can be used for therapeutic purposes. The purpose of this review will provide an overview of NSVs, formulation approaches to improve ocular bioavailability, and its future prospective. The effects of these nonionic surfactants are investigated on their stability and safety to the eye tissues over chronic exposure is provided.

## 2. Nonionic Surfactant Vesicles (NSVs) Considerations

Vesicular systems are lamellar structures made up of amphiphilic molecules surrounded by an aqueous compartment [8–10]. Vesicular systems are useful for the delivery of both hydrophilic and hydrophobic drugs which are encapsulated in interior hydrophilic compartment and outer lipid layer, respectively. Vesicular systems not only provide sustained and controlled release of the medication at the corneal surface but also prevent metabolism of the drug at tear/corneal epithelium surface by various enzymes including esterases and oxidoreductases [11, 12]. Though NSVs may be administered as an eye drop, it is superior to other ophthalmic drug delivery systems in terms of localizing and maintaining drug activity at its site of action. NSVs are lipid vesicles which can increase the rate and extent of drug absorption by encapsulation and easy transition through ocular barrier.

**Figure 2:**
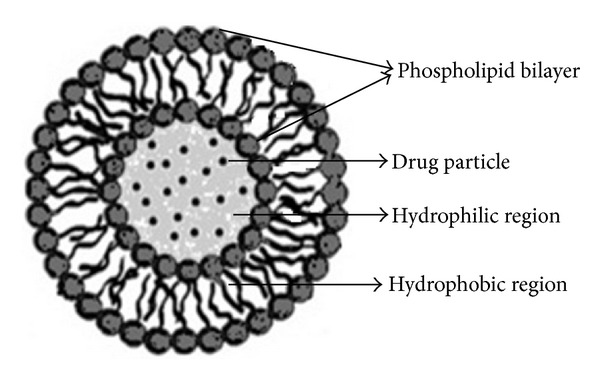
Structure of niosome.

NSVs were first reported in the seventies as a feature of the cosmetic industry [13, 14]. From the early 1980s, these vesicles have gained wide attention by researchers for their use as drug delivery carriers. Niosomes are usually within the size range of 10 nm to 1000 nm. Niosomes are self-assembled nonionic surfactant based multilamellar or unilamellar vesicles in which aqueous solutions of solutes are entirely enclosed by a bilayer membrane resulting from surfactant macromolecules [10, 15] (Figure 2). It acts by reducing the systemic drainage and improving the residence time, which further enhances the ocular bioavailability of drug. The main components of niosomes are nonionic surfactants and the additives (cholesterol and charged molecules). The nonionic surfactants form the vesicular layer and cholesterol improves the rigidity of the bilayer which affects bilayer fluidity and cell permeability. Cholesterol decreased the floating and increased the sedimentation behavior of vesicles. Niosomes in topical ocular delivery are preferred over other vesicular systems because of the following reasons: (1) chemical stability; (2) low toxicity because of their nonionic nature; (3) handling surfactants with no special precautions or conditions; (4) the ability to improve the performance of the drug via better availability and controlled delivery at a particular site; (5) being biodegradable, biocompatible, and nonimmunogenic [16]. They released the drug independent of pH resulting in significant enhancement of ocular bioavailability. Positively charged niosomes also show better binding with corneal surface and more bioavailability [17]. Drug loaded nonionic surfactant vesicles (DNSVs) constitute a versatile drug delivery system, with the ability to overcome physiological barriers and guide the drug to specific cells or intracellular compartments either by passive or ligand-mediated targeting mechanisms [8–10].

DNSVs of various sizes such as polyoxyethylene alkyl ethers [18], series of Spans and Tweens [19], and ester linked surfactants, Brij [20], have been developed in order to improve ocular drug delivery and tested in various animal models. NSVs have been investigated for ophthalmic drug delivery because it can enhance the permeation of poorly absorbed drug molecules by binding to the corneal surface and improving residence time. In addition, niosomes can improve pharmacokinetic profile, enhance therapeutic effect, and reduce toxicity associated with higher dose.

The innovative approach of NSVs in ocular drug therapeutic has been developed by the assessment of the toxicity of ophthalmic formulations and the potential for ocular irritation. Niosomes can be used in drug targeting to various organs such as skin [21], liver [22, 23], lung [24, 25], and ocular systems [26, 27] due to their very small volume which can pass through the narrowest capillaries. They can remain in the blood stream for prolonged period because of their ability to avoid the phagocytes, and as a consequence, they are amenable to controlled and continuous release properties. NSVs have been intensively investigated to deliver a variety of small and large chemical entities like drugs, polypeptides, proteins, vaccines, and genes for improved utilization and reduced toxic side effects.

## 3. Formulation Approaches to Improve Ocular Bioavailability

Various approaches have been explored to improve the ocular bioavailability and the therapeutic duration action of ocular drugs in order to overcome the problems of conventional ocular therapy, such as short residence time, drug drainage, and frequent instillation. Topical bioavailability can be improved by two ways, namely, by maximizing corneal drug absorption and by minimizing precorneal drug loss and the use of drug delivery systems, which provide the controlled and continuous delivery of ophthalmic drugs to the pre- and intraocular tissues.

Conventional drug release approaches such as eye drops, ointments, and aqueous suspensions can be replaced by using a controlled release ocular drug delivery system like in situ gel [28–30], ocuserts [31], collagen shields [32, 33], implants systems [34], and so forth to improve ocular bioavailability and a more controlled, sustained, and continuous drug delivery but the limitations of these systems include patient incompliance, difficulty with self-insertion, foreign body sensation, and inadvertent loss from the eye. Other approaches involve viscosity-increasing polymers which improve the bioavailability of the applied drug by increasing the contact time between the drug and the ocular surface. Colloidal drug delivery systems like mucoadhesive particulates, pH responsive particulates, nanoparticles, microspheres, liposomes, niosomes, and so forth improved the precorneal residence time of the drug and minimized adverse effects. Upon administration into the eye, the particles reside at the delivery site and the drug is released from the particle through diffusion, chemical reaction, polymer degradation, or ion-exchange mechanism [35, 36]. Saettone et al. [37] indicated that the retention of drug in the precorneal tear film is not strictly related to the viscosity of the vehicle but rather to the surface spreading characteristics of the vehicle and to the ability of a polymer to drag water as the vehicle spreads over the ocular surface with each blink. It has been reported that acetazolamide and timolol maleate loaded niosomal formulations showed prolonged decrease in intraocular pressure (IOP) in rabbit [38–40]. Nonionic surfactant-based discoidal niosomes (discomes) of timolol maleate have been reported to be promising systems for the controlled ocular administration of water-soluble drugs; having large size can prevent their drainage into the systemic pool and disc shape could provide better fit in the cul de sac of the eye which leads to their longer retention in the eye [41].

The innovative approach like penetration enhancers has been suggested to improve ocular drug bioavailability either in increasing the permeability of cell membrane or loosen the tight junctions or both [42]. The transport characteristics across the cornea can be maximized by increasing the permeability of the corneal epithelial membrane. Large numbers of enhancers, like actin filament inhibitors, surfactants, bile salts, chelators, and so forth, have been used. It has been reported that digitonin as nonionic surfactant has ability to permeate membranes in a wide variety of cells. In vitro studies revealed an increase in the corneal absorption of a series of polyethylene glycols with different molecular weights [43]. A further approach was the implementation of the mucoadhesive concept to optimize the ocular dosage form. Due to interactions with the mucus layer or the eye tissues, an increase in the precorneal residence time of the preparation was observed. Some mucoadhesive polymers showed not only good potential to increase the bioavailability of the drug applied, but also protective and healing properties to epithelial cells [44]. Commonly mucoadhesive polymers used in ocular therapy are hydroxypropylcellulose, carboxymethylcellulose (CMC) polyacrylic acid, polyethylene glycols, dextrans, chitosan, carbopol, and so forth. Mucoadhesive polymers like chitosan and carbopol-coated niosomal formulation of the encapsulated drug improve the bioavailability [38, 40]. Davies et al. [45] demonstrated that the mucoadhesive polymer-coated (carbopol 934P and carbopol 1342) vesicles significantly enhanced precorneal retention compared to noncoated vesicles only at pH 5 which are assessed by photon correlation spectroscopy and microelectrophoresis technique.

Surfactants have been suggested to increase drug permeability through the cell membranes or via transcellular pathway. When present at low concentrations, these surfactants are incorporated into the lipid bilayer, forming polar defects which change the physical properties of the cell membranes. When the lipid bilayer is saturated, mixed micelles begin to form, resulting in the removal of phospholipids from the cell membranes and also membrane solubilization. Therefore it has been suggested that a dose-dependent increase in the permeability of the cell membrane is responsible for surfactant-induced increases in permeability across different epithelia [42]. Marsh and Maurice [46] evaluated the effect of nonionic surfactants of various hydrophilic lipophilic balance (HLB) values on corneal permeability and toxicity in humans. Surfactants having HLB values between 16 and 17, which correspond to polysorbate 20 and Brij 35, were found to be most effective in increasing corneal permeability. Saettone et al. [47] have investigated the corneal permeability of atenolol, timolol, levobunolol, and betaxolol, the topical *β*-adrenergic receptor antagonists for the treatment of glaucoma, in the presence of various polyoxyethylene alkyl ethers (Brij 35, 78, and 98, resp.). Vitamin E TPGS (d-*α*-tocopheryl polyethylene glycol 1000 succinate), a derivative of vitamin E in a soluble form, has been reported as a potent and effective oral absorption enhancer among the nonionic surfactants [48, 49]. TPGS enhances the drug permeability by P-glycoprotein efflux inhibition. Efflux pump like P-glycoprotein has been recently identified in cornea and corneal cell lines are known to be a major barrier to drug delivery. However, in TPGS the exact inhibition mechanism remains not clearly understood. The mechanism of inhibition may be due to the induced alteration of membrane fluidity which was observed at a surfactant concentration of more than 100 times that needed to obtain a full efflux deactivation [50].

## 4. Physicochemical Properties of NSVs

Colloidal carrier systems like NSVs differ from macroscopic systems because of submicron properties such as high surface area and gel-liquid crystal transition enthalpy energy, stability, and flexibility in their structural characterization, for example, in their composition, fluidity, and size. Size and charge of vesicles have a significant effect on their stability and drug encapsulation. Zeta (*ζ*) potential analyzer determines surface charge of vesicles [8]. Manosroi et al. [51] found that the size of niosomes loaded with gallidermin was smaller for anionic niosomes than cationic niosomes due to neutralization of their negative charge by the positive charge of entrapped gallidermin.

Nonionic surfactants are comprised of both polar and nonpolar segments and possess high interfacial activity. The formation of bilayer vesicles instead of micelles is dependent on the HLB of the surfactant, the chemical structure of the components, and the critical packing parameter (CPP). Nonionic surfactants are preferred due to less irritation power which decreases in order of cationic > anionic > ampholytic > nonionic. Niosomes >10 *μ*m are suitable for drug administration to eye. Nonionic surfactants can be categorized according to the HLB system, the higher the percentage weight of polyethylene oxide in the molecule, the higher HLB value a surfactant holds and the more soluble in aqueous solution [52, 53]. Smaller particles are less stable due to greater surface tension because these require a higher input of energy and thus contain more excess energy and an inherently greater instability than the larger niosomes [10]. Water-soluble surfactants like Tween 20, Tween 80, Cremophor EL, and poloxamer 108, and so forth entrapped in niosomes an increased ocular bioavailability because surfactants act as penetration enhancers which can remove the mucus layer and break junctional complexes [42, 54–56]. Surfactants with an average alkyl chain length of C12–C18 are suitable for the preparation of niosomes [57]. Addition of cholesterol molecule to niosomal system provides rigidity to the membrane and reduces the leakage of drug from niosome [58]. The physicochemical properties of encapsulated drug influence charge and rigidity of the niosome bilayer.

Additionally, particle size distributions of NSVs may be characterized by using laser light scattering method. Blazek-welsh and Rhodes [59] reported that particle size distributions slightly changed with carrier type and total surfactant loading in the niosomal suspension. Hu and Rhodes [60] concluded from the particle size analysis that the conventionally prepared niosomes were larger and slightly more heterogeneous than proniosome derived niosomes. However, sizes distributions were approximately the same but average particle size of conventional niosomes were 2 times more than that of the proniosome derived niosomes. Furthermore, shape and structure of NSVs may be characterized by polarized light microscopy, cryo-scanning electron microscopy (Cryo-SEM), confocal laser scanning microscopy (CLSM), and freeze-fracture transmission electron microscopy. Abdelkader et al. [61] prepared the naltrexone hydrochloride (NTX) loadedniosomes for ocular delivery using the thin film hydration method and characterized by polarized light microscopy and cryo-scanning electron microscopy (Cryo-SEM) to determine the shape of the vesicles. They reported that the prepared niosomes were spherical in shape with smooth surfaces and the onion-like or rose-like folded multilayers were the indication of multilamellarity of the prepared vesicles. Abdelkader et al. [62] developed NTX loaded niosomes and discomes and analyzed the shape by confocal laser scanning microscopy (CLSM) which were found to be uniform and spherical in shape. Marianecci et al. [63] performed the morphological analysis of beclomethasone dipropionate loaded NSVs by freeze-fracture transmission electron microscopy and showed the maintenance of a vesicular structure in the presence of the drug.

Guinedi et al. [39] reported that the multilamellar niosomes were larger in size than their corresponding unilamellar and oligolamellar reverse-phase evaporation niosomes. Also, the niosomes prepared using Span 60 were larger in size than niosomes prepared using Span 40. Longer saturated alkyl chain of Span 60 compared to Span 40 was responsible for the higher entrapment efficiencies of Span 60 multilamellar niosomes. Furthermore, Maiti and coworkers [64] reported that the average diameter of the brimonidine tartrate loaded nanovesicles gradually increased with the increase in surfactant/cholesterol ratio up to a certain extent. Polydispersity index indicated a bimodal intensity size distribution irrespective of the ratio of Span 60 and cholesterol. Differential scanning calorimetry (DSC) studies have shown the melting endothermic transition of pure drug and optimized formulation at 212.84°C which was very nearer to its reported melting point. But a significant reduction in intensity of the peak was observed in the optimized formulation. Also, X-ray diffraction (XRD) analysis revealed a significant loss of crystallinity of the drug in the optimized formulation and existence of both crystalline and amorphous form.

## 5. Fabrication Techniques of NSVs

Niosomes can be prepared from a variety of nonionic surfactants such as alkyl ethers, alkyl glyceryl ethers, poly oxy ethylene 4 lauryl ether (Brij 30), poly oxy ethylene acetyl ethers (Brij 58), and sorbitan fatty acid esters [4]. Niosomes are prepared generally by hydration of nonionic surfactants using hydration media. Fabrication methods that are described here in brief will bring out wide array of possibilities and its flexible applications. Advantages and disadvantages of NSVs are depicted in Table 1.

### 5.1. Ether Injection

Here, accurately weighed lipids and drug were dissolved in organic solvent and injected slowly into an aqueous phase and maintained at 60°C producing large unilamellar vesicles. The size of niosomes obtained by this method varies between 50 and 1000 nm, which mainly depend on the formulation variables and experimental conditions [10].

### 5.2. Transmembrane pH Gradient

Surfactant and cholesterol at a ratio of 1 : 1 were paced into 100 mL round bottomed flask and dissolved in chloroform and evaporated under reduced pressure by a rotary evaporator to produce a thin lipid film that were deposited on the wall of rotary flask. Then, the thin film was hydrated with acidic solution (citric acid) and vortexed in order to mix. After that an aqueous solution of drug was added and again vortexed in order to obtain the final product [65].

### 5.3. Lipid Layer Hydration/Thin Film Hydration

In this method surfactant and cholesterol were dissolved in organic solvents and evaporated under reduced pressure by a rotary evaporator to produce a thin lipid film that was deposited on the wall of rotary flask. The prepared thin film was hydrated with an aqueous solution of drug at a temperature slightly above the phase transition temperature of the surfactants for a specified time with constant mild shaking to obtain multilamellar vesicles [66].

### 5.4. Reversed Phase Evaporation

The surfactants and cholesterol (1 : 1 ratio) were dissolved in a mixture of ether and chloroform. To the resulting mixture, an aqueous phase containing the drug was added, homogenized, and evaporated under reduced pressure in order to remove the organic phase to form viscous niosomal suspension. Further, this was diluted with phosphate buffer saline in order to obtain niosomes [65].

### 5.5. Microfluidization

In this method, two fluidized streams interact at high velocities and were pumped under pressure from a reservoir through the interaction chamber. From the interaction chamber, the streams were passed through a cooling loop to remove the heat produced during microfluidization and returned to the reservoir for recirculation and the process continued until a desired vesicles size was not produced [67].

### 5.6. Bubbling of Nitrogen

This method occurs without use of organic solvents for the preparation of niosomes. Here, cholesterol and surfactant are dispersed together in a buffer and placed into a round bottom flask containing three necks. Nitrogen gas was passed through dispersion mixer with high shear homogenizer resulting in formation of large unilamellar vesicles [68].

### 5.7. Formation of Niosomes from Proniosomes

Proniosomes are a dry formulation using suitable carrier like sorbitol and maltodextrin coated with nonionic surfactants and can be converted into niosomes immediately before use by hydration. Blazek-walsh and Rhodes [59] reported that proniosome derived niosomal formulation provides flexibility and allowed for the optimization of drug encapsulation in the final formulation based on the type and amount of maltodextrin.

## 6. Therapeutic Significance in Ocular Delivery

Various drug delivery strategies mentioned above offer numerous advantages over conventional drug therapy but not overcoming of pitfalls like poor patient compliance and difficulty of insertion as in ocular inserts, tissue irritation, and damage caused by penetration enhancers and collagen shields and change in pharmacokinetic and pharmacodynamics of the drug caused by altering the chemical structure of the drug (prodrug approach). NSVs have significant therapeutic applications in drug delivery and can affect physical properties of drug products such as viscosity, film spreading, and film strength.

NSVs have been widely used in the ocular drug delivery for the treatment of various disorders and infections such as inflammation, dry eye, allergy, ocular hypertension, and glaucoma. Vesicular systems provide prolonged duration of action at the corneal surface by preventing ocular metabolism by enzymes in the lachrymal fluid. Allam et al. [69] reported that acyclovir loaded niosomes were effective for the treatment of herpes simplex keratitis, a condition that can lead to blindness. Bioavailability of ofloxacin niosomal formulation in the eye was improved to 73.8% and in vitro studies showed that these formulations have better residence time and longer duration of action.

Karthikeyan and Pandey [70] prepared diclofenac sodium containing niosomes by using lipid film hydration technique. In vivo studies indicated that Span 60 based niosomes were shown to improve the ocular bioavailability of diclofenac sodium for the prolonged period of time and exhibited no ocular irritation effects. Raghuwanshi et al. [71] have investigated the niosome encapsulated levofloxacin for ophthalmic delivery. In vitro studies indicated that niosomal formulations have exhibited a high retention of levofloxacin inside the vesicle and showed no sign of irritation. Saettone et al. [19] reported that niosomes promoted ocular absorption of cyclopentolate, which is essential in pediatric eye examinations. However, in vitro and in vivo studies revealed that vesicles of nonionic surfactants can increase the absorption of cyclopentolate preferentially by changing the characteristics permeability of the conjunctiva and sclera. Kaur et al. [72] reported an improved ocular bioavailability of cyclopentolate encapsulated niosome, with respect to reference buffer solution, indicating that it can be used as an efficient vehicle for ocular drug delivery.

A modified form of niosomes called discomes having large structures (12–16 mm) by the addition of Solulan C24 as nonionic surfactant is used as ocular drug delivery [38, 72]. Vyas et al. [41] developed timolol maleate loaded niosomes and discomes for the treatment of ocular hypotensive activity and observed that discomes entrapped comparatively a higher amount of drug (25% as compared to 14% in case of niosomes). Moreover, an increase in ocular bioavailability was also observed to be approximately 3.07-fold compared to 2.48-fold in case of niosomes with respect to timolol maleate solution. Guinedi et al. [39] studied acetazolamide loaded niosomal formulation which showed a fairly high retention of drug inside the vesicles (~75%) at a refrigerated temperature for up to three months and also produced significantly less intraocular pressure than free drug and basic niosome solution. Aggarwal and Kaur [40] prepared mucoadhesive timolol maleate (TM) loaded chitosan and carbopol coated NSVs or niosomes by reverse phase evaporation method. In vitro studies indicated a polymer coating which extended the drug release up to 10 h, which continued in a sustained manner over a prolonged period, and they showed only limited systemic absorption and side effects. Pepić et al. [73] have investigated micellar solutions of pilocarpine by using triblock copolymer pluronic F127. In vivo studies showed that it can enhance miotic response to single instillation of pilocarpine eye drops compared to an aqueous solution of the drug. Kuwano et al. [74] observed that micellar cyclosporine eye drop formulation affects its ocular distribution in rabbits and is also superior to nonmicellar formulation (control) with respect to the extent of drug penetration across corneal membrane and ocular tissue exposure.

Abdelbary and El-Gendy [75] reported that gentamicin sulfate loaded niosomes can be used over a longer period of time when installed into eye. In vitro studies indicated a high retention of niosomal formulation inside the vesicles in sustained manner as compared to the drug solution and also observed no irritancy on albino rabbits. Aggarwal et al. [38, 76] reported an acetazolamide encapsulated carbopol 934P-coated niosomal formulation which exhibited more tendency for the reduction of intraocular pressure compared to the marketed niosomal formulation (Dorzox). Studies of aqueous humor pharmacokinetics and acetazolamide encapsulated niosomes revealed that the *C*_max⁡_ of the drug from the niosomal formulation was double that of the drug suspension, showing a significant broadening of peak from 80 to 120 min. The concentration remained over 13 mg throughout the period. Abdelkader et al. [61] also prepared naltrexone hydrochloride loaded NSVs or niosomes for the treatment of diabetic keratopathy by using thin film hydration method. In vitro studies indicated that Span 60-based niosomes containing 30% mol/mol cholesterol revealed liquid-gel transition (transition temperature (Tm) and entropy of 43.5°C and 0.82 kcal/mol, resp.). Such transition reflects potential thermoresponsive properties, which is desirable for ocular delivery. However, niosomal formulation possessed better ocular tolerability and less ocular irritation. Patidar and Jain [77] reported that flupirtine maleate loaded niosomes or NSVs improved the low corneal permeability for effective management of trigeminal neuralgia. Kapoor et al. [78] developed surfactant-laden poly-hydroxy ethyl methacrylate (p-HEMA) contact lenses that can release cyclosporine A (CyA) at a controlled rate for extended periods of time and reported that Brij surfactant-laden p-HEMA gels provide significantly increased bioavailability of ophthalmic drugs by using contact lenses (Table 2).

The nanovesicles were developed for brimonidine tartrate with other excipients like carbopol 940 and HPMC K 15 M using film hydration technique. Brimonidine loaded niosomes were therapeutically effective with a long duration of action due to slow and prolonged zero order release of drug. In vitro and in vivo studies revealed that these formulations showed a prolonged intraocular pressure (IOP) lowering activity in albino rabbits compared to the marketed formulation due to the better partitioning of drug between vesicle and eye corneal surface and exhibited no ocular irritation effects [64, 79–81].

## 7. In Vitro Stability and In Vivo Toxicity

Stability and toxicity of NSVs in ocular delivery is still not much explored. Compared to other vesicular systems, niosomes or NSVs are relatively stable structures; some concern has been expressed regarding the stability in vitro and their toxicity in vivo. Surfactants are used in the preparation of niosomes, which may be a cause of toxicity. However, there are virtually no reports available on the in vivo toxicity of niosomes linked with the concentration of ether or esters surfactants used in the preparation of vesicles. Azmin et al. [82] performed first in vivo experiment on drug delivery by means of synthetic nonionic surfactant vesicles and reported that no adverse effects were observed in the experiment carried out. The stability of the niosome dispersion depends on the stability of the encapsulated drug, surfactant, and the structure of the vesicles. The stability of the system is based not only on the aggregation of the vesicles but also on other factors such as osmotic behavior and other size and shape changes of the system [83]. Niosomes are considered thermodynamically stable and can exist in the metastable state [84]. Abdelkader et al. [27] reported the toxicity of nanosized niosomal vesicles encapsulating naltrexone hydrochloride. These niosomal vesicles were studied with the combination of different surfactants or lipids at various concentrations to test the conjunctival and corneal toxicity, hen's egg test-chorioallantoic membrane (HET-CAM), bovine corneal opacity and permeability (BCOP) test, and corneal histopathological test. Four selected niosomal formulations subjected to 10-day-old HET-CAMs were found to be devoid of any irritant effect, whereas sodium cholate (an ingredient) showed some degree of irritation, which have been observed to be eliminated by incorporating it in niosomes. Maiti et al. [64] studied the stability of nonionic surfactant based nanovesicular formulation of brimonidine tartrate and observed that the formulation when stored at refrigerated temperature offered maximum physical stability and also exhibited no ocular irritation.

Nonionic surfactants enhanced the systemic absorption of melanocyte stimulating hormone via the ocular route in rabbits [85]. The cytotoxicity order of surfactants on rabbit corneal epithelial cells was cationic > anionic > nonionic; however, triton X-100 had a ranking similar to anionic surfactants [86]. Poloxalene (30% polyethyleneoxide and 70% polypropylene oxide, MW 3000) inhibited neutral fat and cholesterol absorption in rabbits [87]. The study of the uptake of neutral red by rabbit corneal cells revealed that nonionic surfactants have a lower toxic effect than cationic, anionic, and amphoteric ones [88]. Furrer et al. [89] tested currently used nonionic (Tween 20, Pluronic F127, Brij 35, and Mirj 51), anionic (sodium lauryl sulfate and sodium cholate), and cationic (benzalkonium chloride and cetrimide) surfactants for ocular irritation, in both rabbits and mice, using confocal laser ophthalmoscopy, in which corneal lesions subsequent to instillation of surfactants are specifically marked by fluorescein and assessed by digital image processing. The tests revealed the following irritation rankings: cationic > anionic > nonionic surfactants. Extremely low toxicity of NSVs has been demonstrated in rat after administration both by subcutaneous or intramuscular routes. At doses up to 575 mg/kg body weight there was no persistence of NSVs at the site of injection (s.c.) [90].

## 8. Safety Assessment

Several researchers have investigated that NSVs have a long history of being safe in ophthalmic use. In recent decades, scientists has extensively studied about ocular dosage forms which have recorded the toxicological signs of ocular tissues exposed to topically applied drugs. Ocular tissues, such as the cornea and conjunctiva, are susceptible to injuries and adverse ocular effects, either from the administered drug or excipients used in the finished pharmaceutical product [91, 92]. Maurer et al. [93, 94] have investigated that nonionic surfactants have potential to cause corneal stromal and endothelial cell changes by affecting both corneal and conjunctival epithelium to various degrees. Over time, ocular responses to surfactants were evidenced by the presence of keratocyte regeneration, corneal neovascularization, and conjunctivalization of the corneal epithelium. These responses are accompanied by clinical evidence of irritation such as corneal opacity, conjunctival redness/chemosis, and discharge. The pathobiology of surfactant-induced ocular irritation can only be demonstrated with the new technology of noninvasive, in vivo confocal microscopy (CM). Hazleton [95] reported polysorbate 80 as nonionic surfactant to be nonirritating to the rabbit eye up to a concentration of 10% and has been used in a number of marketed ophthalmic solution drops. Maiti et al. [64] studied a number of common ophthalmic excipients like Span 60 and cholesterol using film hydration technique and dispersed in viscous carbopol solution. It was observed that optimized formulation showed better ocular hypotensive activity and also exhibited no ocular irritation effects observed in the rabbit eye.

## 9. Future Prospective

In this new span of investigation, recent NSVs based drug delivery via ophthalmic route has proved significant advancement for future perspectives in relation to prolonging the preocular retention on the eye surface and to the improvement of transcorneal penetration of novel therapeutic agents such as protein and peptide drugs.

### 9.1. Mucoadhesive Polymeric Systems (MPS)

MPS have capacity to adhere to the mucin coat covering the conjunctiva and corneal surfaces of the eye by noncovalent bonds and improve the bioavailability of the drug. These systems significantly prolong the drug residence time and high drug flux through the absorbing tissue [96]. It has been reported that mucoadhesive (chitosan) coated niosomal system for timolol maleate (TM) showed no significant effect and also possess a better in vitro corneal penetration and IOP lowering effect [97]. Mucoadhesive (carbopol 940) coated nanovesicular system of brimonidine tartrate have been found to provide better ocular hypotensive activity than marketed drops on albino rabbits and exhibited no ocular irritation effects [64].

### 9.2. Drug-Cyclodextrin Complex Approach

In ophthalmic preparations, coadministration of cyclodextrins has been reported to increase corneal penetration, ocular absorption, and the efficacy of poorly water-soluble drugs such as dexamethasone, cyclosporine, and acetazolamide which are attributed to the ability of cyclodextrins to increase the aqueous solubility of lipophilic drugs without affecting their intrinsic ability to permeate biological membranes. It has also been found that cyclodextrin drug complexes can increase the entrapment of drug in nonionic surfactants vesicles and improve the activity [98]. It has been observed that 10% hydroxypropyl-ß-cyclodextrin (CD) loaded acetazolamide niosomal formulation can increase the corneal permeability [99]. Bucolo et al. [100] reported that the indomethacin-hydroxyl propyl methylcellulose (IND-HPMC) treated group showed significantly higher aqueous and retinal levels of indomethacin at each time interval compared with IND-CD treated group which indicated that IND-HPMC formulation has good ocular distribution reaching relevant indomethacin levels in the posterior segment of the eye.

### 9.3. Penetration Enhancer

The use of penetration enhancers in ophthalmic solutions improves both corneal and conjunctival delivery of drugs. Furthermore, addition of these enhancers to the vehicle of ophthalmic solution has been used to reduce the size of drop instilled. Therefore, improved ocular bioavailability and therapeutic response could be obtained [101]. Grześkowiak studied the effect of sodium deoxycholate, poly oxyethylene-9-lauryl ether (nonionic surfactant), and L-*α*-lysophosphatidylcholine on sulfadicramide dialysis through synthetic lipophilic membranes and animal cornea in-vitro. It was observed that ophthalmic ointment containing sulfadicramide and these absorption promoters has been used for the treatment of inflamed eyelid margins, ocular conjunctiva, and the external surface of cornea. Polyoxyethylene-9-lauryl ether was found to be the most effective surfactant among these [102, 103].

## 10. Conclusion

In recent years, several researchers had evaluated the feasibility of nonionic surfactant vesicular carriers as an ophthalmic delivery system to prolong the preocular residence of ocular drugs and improve their bioavailability. Hence, these systems possess a convenient methodology for an ocular drug therapy with wide potential development though controlled clinical studies which are necessary to provide more information regarding their long-term safety and stability. Niosomal formulations can also be used for resolving solubility factor and prolong the duration of exposure of a new drug by increasing retention through mucoadhesion. The potential for the growth of newer nonionic surfactant would serve the purpose of controlled and sustained delivery for treating vision-threatening diseases. NSVs have the potential to target ocular tissues at high therapeutic value offering several favorable biological properties, such as biodegradability, biocompatibility and mucoadhesiveness, which fulfill the requirements for ophthalmic application. Physicochemical characteristics such as size, surface charge, morphology, physical state of the encapsulated drug, drug release properties, and stability of the niosomal systems are of particular importance for topical ocular application.

Furthermore, since there is a lack of medication that can be used to improve in the precorneal retention, transcorneal permeation, and therapeutic efficacy, the NSVs offer distinct advantages over existing ocular drugs. In addition, niosomal formulations have been evaluated for encapsulation of various drug molecules of different therapeutic classes. Newer concepts of exploiting the use of cyclodextrins approaches and mucoadhesive polymers such as chitosan and carbopol in niosomal systems also need to be evaluated for ocular therapy. However, the therapeutic potential for the treatment of bacterial conjunctivitis and glaucoma will need to be assessed in further studies.

## Figures and Tables

**Table 1:** Fabrication techniques of NSVs: advantages and disadvantages.

Method	Advantages	Disadvantages/drawbacks
Ether injection	Process is very simple and mild. Large unilamellar vesicles are formed.	Population is heterogeneous. Relatively low encapsulation efficiency.

Transmembrane pH gradient	Simple, efficient, and economical procedure for active loading of weak amphiphatic drugs.	Low entrapment efficiency and heterogeneous size. Need for secondary processing steps such as extrusion or sonication.
	Applicable for sustained release of noisome encapsulated drugs from ammonium niosomes.	Difficult standardization and low reproducibility.

Lipid layer hydration	Increasing stability. Suitable for large-scale preparation. Large multilamellar vesicles are prepared.	Low internal volume and low encapsulation efficiency. Size distribution is heterogeneous.

Reversed phase evaporation	High encapsulation efficiency.	Exposure of the materials to be encapsulated to organic solvent and to sonication.
	Encapsulates small and large macromolecules.	Toxicity due to organic solvent.

Microfluidization	Greater uniformity, smaller size and better reproducibility.	Low encapsulation efficiency. Leaking of encapsulated drug.
	Large production of lipid vesicles without dissolving the phospholipids in organic solvents.	Has tendency to aggregate or fuse and may be susceptible to hydrolysis and or oxidation.

Bubbling of nitrogen	One-step preparation method without the use of organic solvents.	Instability on prolonged storage.

Formation of niosomes from proniosomes	Easy storage and handling. Suitable for both hydrophilic and hydrophobic drug.	Time consuming method and involves specialized equipment with vacuum and nitrogen gas.
	Minimizes problems of physical stability such as aggregation, fusion, and leaking of entrapped drug.	Amount of unentrapped drug should not be analyzed.

**Table 2:** List of drug loaded nonionic surfactants vesicles in ocular delivery.

Surfactant	Drug loaded	Comments	References
Brij 35, 78, 98, and 700	Atenolol, timolol, betaxolol cyclosporine A	Significantly increased the corneal permeability. Significantly increased bioavailability of ophthalmic drugs by using contact lenses.	[52, 76]

Polysorbate 80, polyoxyl 40 stearate, and polyoxyl 60 hydrogenated castor oil	Cyclosporine A	Improve ocular CsA penetration and are clinically useful in the treatment of immune-mediated ophthalmic diseases.	[71]

Pluronic F127	Pilocarpine	Enhanced miotic response to a single instillation of pilocarpine eye drops compared to an aqueous solution of the drug.	[68]

Polysorbates 60 and 80 and Brij 35	Gentamicin sulphate	Effective in the prolongation of drug release from the ocular delivery and observed no irritancy on albino rabbits.	[72]

Polysorbate 20	Cyclopentolate	Promoted ocular absorption.	[23, 25]

Solulan C-24 and span 60	Timolol maleate	Treatment of ocular hypotensive activity. Exhibited sustained controlled ocular drug delivery.	[45, 46]

Spans 20, 40, 60, and 80	Acyclovir	Effective for the treatment of herpes simplex keratitis.	[67]
	Acetazolamide	Effective in enhancing the bioavailability of drug and lowered the intraocular pressure.	[43, 45, 73]
	Brimonidine tartrate	Improved bioavailability and increased precorneal residence time. Decreased the intraocular pressure for a prolonged period of time and nonirritant effect	[77–80]
	Naltrexone hydrochloride	Possessed better ocular tolerability and less ocular irritation.	[74]
	Fluconazole	Improved permeability as compared to marketed formulation and nonirritant effect.	[25]
	Flupirtine maleate	Improved the low corneal permeability for effective management of trigeminal neuralgia.	[75]
	Diclofenac sodium	Nonirritant and safe vesicular system for the effective ocular drug delivery.	[68]
	Levofloxacin.	Efficient in prolonging the drug release with reduced side effects.	[69]
